# Tuberculosis of the Peritoneum1Read before the Erie County Medical Association, September 8, 1902.

**Published:** 1902-11

**Authors:** William H. Mansperger

**Affiliations:** Buffalo, N. Y., 1003 Main Street.


### Tuberculosis of the Peritoneum.[1]

By WILLIAM H. MANSPERGER, M. D., Buffalo, N. Y.

THIS not uncommon form of tuberculosis usually arises secondarily from tuberculosis of the pleura or the abdominal organs, so that the continuous extension of the process is clearly demonstrable. In some cases, however, this continuous connection of tubercular peritonitis, with a local tuberculosis in the vicinity, cannot be traced. Here, no doubt, the infection has been carried from some distant focus; either through the medium of the circulation, or through that of the lymphatics. It also appears that cases are not infrequent where there are no other demonstrable lesions of the same nature in other parts of the body. In such a case we would consider a direct infection as being probable, but not positive, as in life the source of the infection is often obscure and the original focus only discoverable at autopsy.

Since the pleura and peritoneum are in direct communication through the intercommunication of the lymphatics of the diaphragm, it naturally follows that the disease is often transmitted from the former to the latter; and this is, in all probability, the usual source of origin in tuberculosis of the peritoneum, situated in the neighborhood of the diaphragm.

Tuberculosis of the bowel is also a frequent etiological factor, and is, as a rule, in the adult, secondary to pulmonary tuberculosis; the active bacillus being carried from one organ to the other by the swallowing of the sputum. Primary tuber-

---

1. Read before the Erie County Medical Association, September 8, 1902.

culosis of the bowel, however, is not wanting. In children especially does primary bowel tuberculosis occur and is often the cause of tubercular peritonitis. In these conditions it is likely that the virus is swallowed with the food. The process in the bowel begins in the lymphatics of that organ—namely, in Peyer's patches and in the solitary glands; in the large as well as in the small intestines, the ileocecal region being a favorite seat. From these processes of disease in the bowel the infection is transmitted to the peritoneum, either through a process of ulceration without perforation, but usually through the medium of the retromesenteric glands, which themselves become involved and may be felt as enlarged and hard nodular masses behind the mesentery.

In women the peritoneum frequently is the subject of tubercular infection through extension of the process from the genitals. In tubercular endometritis the avenue of infection is often through the tube into the peritoneal cavity; the uterus and ovaries also frequently show localisation of the process, and the peritoneum may become involved by direct invasion from these points.

The urinary apparatus is likewise a favorite seat of this disease, the kidney and suprarenals being often involved in pulmonary cases through the medium of the circulation. The kidney may also become involved by extension of the process up the ureter from without. The bladder is also a frequent location of primary tubercular disease. Why it is that in women the urinary tract is rarely involved, it is difficult to explain; nevertheless, this seems to be undoubtedly the case. The prostate gland, the seminal vesicles and the testes are all prone to the action of the virus, and are often instrumental in the production of the trouble in the peritoneum.

The original focus may be situated in the skin, the lymphatics, the bones, or in almost any other tissue of the body. As tuberculosis has its origin most frequently in tissues which are subject to external influences and irritations, we naturally look to the lungs, the bowels and skin as the most likely portals of entrance; these organs usually themselves become involved and the peritoneum secondarily from these points. The poison may, however, be carried to distant points without the involvement of the point of entrance.

When the virus gains access to the peritoneum that organ soon becomes studded with miliary tubercles; this may or may not be accompanied by a process of inflammation. The non-inflammatory form of the disease is usually spoken of as tuberculosis of the peritoneum; the inflammatory form, as tubercular peritonitis. In the latter the organ is red, congested and swollen; there is an adhesive agglutination of bowels and neighboring organs, which is everywhere in evidence. In the beginning of the process these adhesions are very easily separated, but in the course of time they become dense and well organised, forming a pseudo-membrane so that no point of separation can be found. In this way the abdominal organs are often distinctly encapsulated by a thickened false membrane. This connective tissue formation, as elsewhere in the body, is followed by contraction and deformity. The mesentery and omentum, on account of their structure, are particularly prone to this process; the omentum often being converted into a hard and thickened cushion, roll or ball; as a result of the contraction and deformity of the mesentery the entire abdominal cavity may be filled with hard and nodular masses.

There is usually a fluid exudate, either serous, hemorrhagic or purulent. The hemorrhagic exudate occurs frequently and is similar to the exudate in hemorrhagic pleuritis. The fluid exudate varies in amount as well as in location; the amount may be very small or it may be enormous; it may be free or it may be localised between the agglutinated loops of intestines.

Another condition which is not only interesting pathologically but is also instructive as to symptoms and diagnosis, is the not infrequent occurrence of cirrhosis of the liver in the advanced stages of this disease.

With the gross pathology so distinct and clearly defined, the inference of an equally distinct symptomatology and clear diagnosis would naturally suggest itself. This, however, is not the case, in a goodly proportion of instances, and particularly so in tubercular disease of the female pelvic organs, for here we cannot exclude other pathological conditions with the same amount of certainty as is the case where the disease occurs in the abdominal cavity proper. It should, not however, be inferred that the diagnosis is, as a rule, difficult or impossible; on the contrary, the diagnosis can, generally, be made with as much certainty as in most other diseases, and very often is so simple that it could scarcely be mistaken for anything else. It is only in cases of tuberculosis without evidence of inflammation that the diagnosis becomes to a certain degree impossible.

The symptoms upon which most reliance can be placed in this disease are abdominal pain and tenderness, abdominal enlargement, either general or circumscribed, tumor and fever. The pain is, as a rule, dull and aching, rarely intense, and is circumscribed. Tenderness on pressure is present in all cases, but is rarely very marked, the sensation being one rather of soreness than of acute tenderness.

The general abdominal enlargement may be so pronounced as to resemble ascites or a large ovarian cyst. Here the method of exclusion in the diagnosis becomes very valuable. Our first thought would be to exclude ascites. In ascites we have a number of characteristic symptoms which will rarely lead us astray. We look for bulging of the flank and flattening of the abdomen; where there is free fluid, for dulness on percussing the flank, and for tympany over the abdomen when the intestines are provided with sufficient length of mesentery to permit them to float to the surface. When we are dealing with an ovarian cyst or an encysted tubercular peritonitis, the very opposite holds true; the flanks are contracted, the abdomen prominent and projecting; there is tympany over the flank where the colon is fast through adhesions, and dulness over the prominent portion of the abdomen, because here the prominence is produced by a circumscribed collection of fluid. In ascites we would also look for previous symptoms of contracted liver, gastric hemorrhage, enlarged spleen, edema of the lower limbs and genitals. Having succeeded in excluding ascites we come to differentiate between ovarian cyst and encysted tubercular peritonitis. I believe that inspection alone would be able to decide this problem for us; as in the case of a cyst which is always tense, the overlying abdominal wall is splinted and does not move with the free parts of the abdomen during expiration and inspiration; an encysted distention being less tense and more or less compressible readily permits the movements of respiration to take place.

In the case of hard, circumscribed tumors, with more or less fluid accumulation, we are again confronted with a differential diagnosis between this disease and malignancy of the peritoneum. Here the difference in the character of the pain—sharp and acute in malignancy, dull and aching in tuberculosis,—will be of assistance to us. More important still is the deportment of the temperature; for instance, subnormal morning temperature followed by a rise in the course of the day, especially if accompanied by sweats and chills, would prove of great value. We would also look for evidence of tuberculosis in other organs.

There are also a number of other symptoms which point quite strongly to the existence of this disease; the abdomen is usually not only distended, but it is unevenly so, some links of bowel being more inflated than others, on account of kinks and adhesions. Palpation at times gives characteristic results, when mesentery and omentum are thickened, hard and deformed, and when some bowel links are more thickened and agglutinated than others, thus producing a field of uneven resistance which can be mistaken for no other condition except malignancy, and this is not very likely. Another condition pointing to the existence of this disease is the presence of a number of localised collections of fluid, which can be distinguished by their fluctuation, dulness on percussion, and from the fact that the fluid does not gravitate to other parts when the position of patient is changed. There may be circumscribed dulness without fluctuation, *i. e.*, without fluid; this is caused by thick fibrinous exudates, and dense agglutinations. A very important condition, and one which I have seen several times, is cirrhosis of the liver; the organ first undergoing enlargement, afterwards contraction. A friction sound over the liver region may also at times be heard.

Diarrhea is often present and is the rule when the trouble is secondary to tubercular disease of the bowel. Constipation is not rare, and there may be partial obstruction when there is much kinking in the bowels. Urinary symptoms only become prominent when the bladder is involved, and this is the rule in pelvic cases. The first symptoms often make their appearance in the right iliac region, owing to the fact that the ileocecal region is a favorite seat of localisation; under these conditions the disease may be mistaken for appendicitis. A positive differential diagnosis would here be impossible, but may be surmised when the patient is suffering with tuberculosis of some other organ.

The female genitals are perhaps more frequently the primary seat of this disease, than any other organ in the abdomen. The reason for this can probably be explained by the frequent occurrence of inflammation in these parts following labor and miscarriage, thus creating a weak point of resistance to the tuberculous material carried there through the medium of the general circulation.

Two other important factors in the diagnosis are the temperature and pulse; lasting hectic with continuous high pulse rate makes the existence of tuberculosis probable.

If puncture is undertaken and the fluid found to be either fibrinous or hemorrhagic the symptom would be quite characteristic. It is always important to look for evidence of tuberculosis elsewhere in the body, because the peritoneal infection is usually secondary in the nature of its origin.

What should be the treatment when the diagnosis has been established? It appears that any effort which we are able to make to influence .this disease favorably, apart from surgical interference, is very limited. Aside from the very important constitutional treatment, such as diet, fresh air and rest, nothing but the relief of symptoms remains to be accomplished with drugs. Creosote, guaiacol, cinnamic acid and other drugs which have gained favorable reputations in the treatment of this disease in the lungs give no promise of result or success here. This can also be said of tuberculin. We have the alterative, iodine treatment, and the antiphlogistic mercury treatment, to which we may pin our hopes, and it is possible that we may be able to achieve some results in a few cases, in the very early stages; but where the disease is at all advanced, with the patient's digestive and bowel functions in poor condition, and the disease making daily progress, it is not likely that we would adopt this form of treatment with much hope or ardor, for such treatment requires a long period of time for its proper trial and such time is valuable in a patient with so deadly a disease; so valuable that the patient may be beyond the possibility of help through surgical treatment which alone holds out much hope for success or improvement. Whether or not the surgical treatment is ever followed by a complete cure is difficult to say, for there is a great diversity of opinion on that question. On the other hand, there can be no question as to the great and almost immediate improvement which in the large majority of cases follows its employment. That the ultimate prognosis in this disease, when complicated with pulmonary tuberculosis—and unfortunately such is often the case—is dark, very gloomy, perhaps; nevertheless, the patients may often be so much improved, and their lives so much prolonged that we should always stand ready to give them their only chance. We should, of course, make sure that the patient's strength is of sufficient quality so as to give good promise of withstanding the shock, which is not very great. In such patients the immediate danger is very small. Why it is that laparatomy so favorably influences this condition has not as yet been satisfactorily explained in a scientific manner. The fact, however, remains that it is usually followed by the complete disappearance of all fluid exudate and a general improvement in all the symptoms. It is true that a recurrence of the process may take place in a comparatively short time; in others, the improvement covers a period of years, and may be permanent.

The technique is usually simple as compared with other operations in the abdomen, as often nothing can be done except to flush out with normal salt solution and to close. If the focus from which the process has spread can be removed without endangering the integrity of intestines and other viscera, it should be done. The most favorable location for this is in the uterus, tubes and ovaries, and these organs when diseased should always be removed when the risk is not greatly increased thereby, because they usually contain broken down foci which may later act as sources of reinfection. Drainage is only employed in the presence of cavities whose contents have undergone secondary infection.

I ask permission to report a small group of eleven personal cases, all of which were treated by laparatomy. Of these, three were males; eight females; in two males and in three females the process was localised; in the other six, generally disseminated over the entire peritoneum. Of the five, in which the process was localised, four are alive and in good health; two, four years after operation; two, two years, and one, one year. Of the six in which the process was general on the peritoneum, three are alive, and three are dead. Of the three alive, two had recurrences one year and a half after operation; one is in good condition, one year after operation. In four of the eleven, lung disease was undoubtedly present. Of these four three died of pulmonary tuberculosis, one is alive and an invalid; three of the four were relieved of the abdominal symptoms, and were much improved after the operation.

To summarise: in good health, five; died, four; invalids, two. What I have seen, as well as what I have heard and read, all prompt me to enter a plea for the early surgical treatment in tubercular disease of the peritoneum.

1003 MAIN STREET.

